# Olfactory asymmetric dysfunction in early Parkinson patients affected by unilateral disorder

**DOI:** 10.3389/fpsyg.2015.01020

**Published:** 2015-07-16

**Authors:** Gesualdo M. Zucco, Francesco Rovatti, Richard J. Stevenson

**Affiliations:** ^1^Department of General Psychology, University of Padova, Padova, Italy; ^2^Department of Psychology, Macquarie University, Sydney, NSW, Australia

**Keywords:** Parkinson patients, olfaction, odor identification and odor recognition tasks, monorhinic presentation, asymmetric olfactory deficits

## Abstract

**Introduction:** Parkinson’s disease (PD) often first presents with asymmetric motor symptoms. A number of studies have now established that sensory deficits can also be similarly asymmetric. It is well established that PD is associated with marked olfactory dysfunction, but whether this too present asymmetrically is a currently contentious question.

**Methods:** To address this, we recruited 12 early stage Parkinson patients with right-sided motor symptoms and compared them to 12 healthy age-matched controls on tests of olfactory identification and recognition, administered separately to each nostril.

**Results:** Data analyses indicated that Parkinson patients performed worse with the left nostril on both tasks, while no nostril-related differences were observed for the healthy age-matched control group on the same comparisons.

**Conclusion:** These findings support the idea that asymmetric deficits do extend into olfactory performance in PD—as they do into other sensory domains—and we examine the possibility that they might be a particular feature of right-sided motor symptom presentation.

## Introduction

Parkinson’s disease (PD) typically manifests asymmetrically (Gelb et al., 1999). Indeed, the presenting asymmetry of tremor, bradykinesia and rigidity is a differentiating feature of PD relative to other related conditions (e.g., Adams and Salam-Adams, 1986; Brooks and Pavese, 2009). Even as the disease progresses, and bilateral presentation of motor symptomology appears, asymmetry is still readily evident (Djaldetti et al., 2006). Asymmetric presentation of clinical symptoms in PD is not limited to just motor-related phenomena. A number of studies have now documented effects that extend into the sensory domain. These include poorer capacity for sound localization in PD patients (Lewald et al., 2004), asymmetric differences in pain sensitivity and perception (e.g., McNamara et al., 2010), and in bodily fatigue (Friedman and Friedman, 2001). In the present report, we examine whether asymmetries also occur in the olfactory system in PD patients.

Parkinson’s disease patients exhibit deficits in the detection, recognition and identification of odors (Ward et al., 1983; Quinn et al., 1987; Zucco et al., 2001; Doty, 2003, 2012; Hawkes, 2003; Huisman et al., 2004; Katzenschlager and Lees, 2004; Kovacs, 2004). Few studies have examined whether these various olfactory deficits are bilateral or whether they are asymmetric, and if they are asymmetric, whether they are ipsilateral or contralateral to motor-related impairments. In one study, Doty et al. (1992), examined groups of medicated and unmedicated PD patients, who completed a standardized smell identification task. As with other reports in the literature (see Doty, 2012) there was a significant impairment in odor identification performance in the PD patients when compared to a group of healthy age matched controls, with medication status in the PD patients having no effect on olfactory test performance. More pertinently here, there was no evidence of asymmetry in identification performance. In contrast to this finding, a small study by Zucco et al. (2001), examining olfactory recognition and identification in six PD patients with predominantly right-sided motor symptoms found significant left-sided impairment on the recognition task, relative to a group of healthy age matched controls. In the current study, we wished to extend Zucco al.’s (2001) original finding, by determining whether a similar asymmetry in olfactory functioning could be found on both tasks in a larger group of early stage PD patients, with right-sided motor symptoms.

## Materials and Methods

### Participants

Two groups were formed, one consisting of 12 Parkinson patients aged on average 65.3 ± (SD) 4.8 years (range, 55–70 years), matched for age and gender to 12 Normal controls aged on average 69.1 ± (SD) 5.1 years (range, 59–76 years). Both sexes were equally represented. All participants were right-handers, as ascertained by their responses to the Oldfield’s (1971) questionnaire for handedness.

The patients, at an early stage of the disease (*M* duration of symptoms 2 years and 3 months) were examined at the Neurological clinic of the St. Raffaele hospital in Milan (I). The severity of their dysfunction was evaluated from 1 to 1.5 on the Hoehn and Yahr (1967) and Fahn and Elton (1987) scale. The disorder was unilateral with only the right side of the body affected. The patients were treated at the time of testing with Sinemet (L-Dopa with carbidopa) and Requip (Ropirinolo).

Both groups of participants were in good health and free of major medical illness and signs of dementia (as ascertained on the basis of their scores on the Mini Mental State Examination). Also, they had no history of major olfactory pathologies or drug use, which may affect olfactory function. Exclusion criteria (see, e.g., Zucco and Bollini, 2011) were related to conditions causing temporary or permanent alterations to the sense of smell (e.g., current allergic rhinitis, polyposis, viral infection, nasal trauma, head injury), neurological disorder that might affect cognitive function, drug or alcohol abuse, and smoking habits (only non-smokers were tested).

Informed consent was obtained from the participants in the study. The study was conducted in accordance with the Declaration of Helsinki for experimentation with human subjects and approved by the local committee.

### Materials and Procedures

The general procedure was the same as that used in other published papers (see, e.g., Zucco and Bollini, 2011).

Participants underwent an odor identification and an odor recognition task of suprathreshold common odorants comparable for subjective intensities, as based on prior laboratory comparisons. Some of the odorants were drawn from household items (e.g., anchovy paste, shoe-polish), while others were essences and essential oils (Kart laboratories, Lausanne, Switzerland) with the remainder drawing upon stimuli from the “Sniffin’ Sticks” test kit (Burghart Instruments, Wedel, Germany). Ten odorants served as target and 30 as distracters (see Table 1, for details). Excepting the Sniffin’ Sticks the odorants were either dissolved in mineral oil, in distilled water or presented neat, and placed in small test glasses (height: 15 cm, diameter: 1.3 cm) fitted with rubber lids connected to a cotton swab wrapped around the end of a stick. These substances were refreshed every 48 h which was deemed sufficient to maintain a fairly constant subjective intensity.

**Table 1:** **Odorants used listed in alphabetic order and their respective concentration**.

**Number**	**Targets**	**Distracters**		
1.	Almond (100% N)	Anchovy paste (100% N)	Jasmine (70% MO)	Shoe-polish (100% N)
2.	Cinnamon (SS)	Banana (SS)	Juniper (100% N)	Sulfur (100% N)
3.	Coffee (SS)	Boot grease (100% N)	Lemon (SS)	Strawberry (100% N)
4.	Garlic (SS)	Camphor (100% N)	Licorice (SS)	Tar (100% N)
5.	Ink (100% N)	Chocolate (SS)	Mustard (75% DW)	Tobacco (100% N)
6.	Lavender (SS)	Clove (SS)	Onion (80% DW)	Tomato (100% N)
7.	Grappa Liquor (100% N)	Denatured alcohol (70% DW)	Paint (100% N)	Turpentine (SS)
8.	Mint (SS)	Dish washing liquid (100% N)	Petrol (70% DW)	Vanilla (100% N)
9.	Oregano (100% N)	Fennel (100% N)	Pine (100% N)	Vinegar (70% DW)
10.	Rose (SS)	Honey (100%N)	Rum (100% N)	Violet (100% N)

DW, dilution in distilled water; MO, dilution in mineral oil; N, neat; SS, “Sniffin’ Sticks.” Percentage indicated the quantity of odorant used.

For both the recognition task and the identification task, participants were instructed to keep their eyes closed for the entirety of each test. On the recognition task each trial comprised the presentation of a target odorant followed by a recognition set of four odorants. Each participant smelled the target—randomly chosen from the set of 10—for about 4-s. About 3–4 s later the participant was presented, one at a time, with four test tubes one of which contained the previously sniffed odorant, and he/she was asked to recognize the target. The distracter stimuli on each recognition trial were randomly selected from a pool of distracters (see, Table 1). A 6-s interstimulus interval separated odor presentations so as to avoid carry-over adaptation effects. Between trials a 20–25 s rest was provided. On the identification task the participant had to smell each odorant for 4 s, drawn at random from the set of 10, while the examiner read aloud four alternative verbal labels (see, Zucco et al., 2014). Each participant had to identify the correct label for the odorant. On both the recognition and identification tests the stimuli were delivered separately to each nostril, with the other covered by a cotton swab. The distance between the stimulus and the subject’s nose was kept constant (i.e., the odorants were kept approximately 2 cm in front of the nostrils). The experiment took place in one quiet, well-ventilated room. The order of tasks and the order of the nostril exposed to the odorants was counterbalanced among participants. Responses were scored for accuracy. The participants’ scores ranged from 0 to 10. Each test procedure lasted about 20 min per participant.

### Experimental Design and Statistical Analyses

Results were analyzed by means of SPSS 21 statistical software for windows.

Number of correct responses were submitted to a three-way mixed-design analysis of variance (ANOVA), with “Groups” (Parkinson patients vs. Controls) as between factor, and “Nostril” (Left vs. Right) and “Tasks” (Recognition vs. Identification) as within factors.

## Results

**FIGURE 1:**
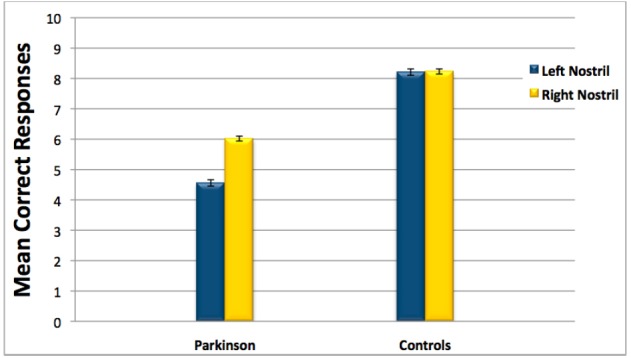
**Overall performance on the olfactory tests (combined recognition and identification scores) for Parkinson patients and healthy age-matched controls as a function of nostril stimulated.** Error bars represent standard errors.

Overall performance on the olfactory tests (combined recognition and identification scores), as a function of nostril stimulated, are depicted in Figure 1. The gender of the participants was not included as a variable in the following analyses, since it had no influence on tests performance as ascertained by a (unreported) set of prior analyses.

The analysis revealed a significant effect of factors Groups: [*F*(1,22) = 61.2, *P* = 0.0001] and Nostril: [*F*(1,22) = 7.48, *P* = 0.012] and the interaction Groups × Nostril [*F*(1,22) = 8.51, *P* = 0.012], suggesting that performance differed between nostrils in the Parkinson patients to a greater extent than for controls (see Figure 1). To confirm this interpretation we conducted a pair-wise *post hoc* comparison using the Tukey test, which showed significantly worse performance with the left nostril compared to the right only in the Parkinson patients group (*P* = 0.01). The mean overall performance scores for each group by nostril stimulated were as follows: Parkinson patients (Left nostril: *M* = 4.56, SEM = 0.4; Right nostril: *M* = 6.02, SEM = 0.3); Controls (Left nostril: *M* = 8.21, SEM = 0.2; Right nostril: *M* = 8.23, SEM = 0.3).

## Discussion

Consistent with previous reports, the PD patients as a whole were significantly poorer on our olfactory identification and recognition tasks than the healthy age matched controls (Ward et al., 1983; Quinn et al., 1987; Zucco et al., 2001; Doty, 2003, 2012; Hawkes, 2003; Katzenschlager and Lees, 2004; Kovacs, 2004). However, while the consensus (e.g., Doty, 2012) position would seem to be that these deficits should be bilateral, our findings would seem to indicate otherwise. We found significant left-sided impairments in the PD patients on both olfactory recognition and identification tasks, when contrasted to our healthy age matched controls. Not only does this result extend the previous preliminary findings of a similar left-sided olfactory deficit in PD patients with right-sided motor symptoms (Zucco et al., 2001), it also provides further evidence that olfactory deficits in PD can be asymmetric—as with certain other sensory deficits tested in this disorder (e.g., Friedman and Friedman, 2001; Lewald et al., 2004; McNamara et al., 2010).

One obvious question to arise from these findings is why we have observed asymmetries in olfactory performance that were not seen in Doty et al.’s (1992) original study. It has been suggested that PD may well represent a cluster of related but actually quite distinct disorders (e.g., Sian-Hulsmann et al., 2011). One feature of PD where this distinction has been made before relates to the presenting side of the disorder. The literature suggests that initial right-sided presentation may be associated with particular features including reduced PD severity and a slower rate of progression (Riederer and Sian-Hulsmann, 2012), as well encompassing other disease-related differences (e.g., reduced cognitive deficits; Huber et al., 1992). We tentatively suggest that initial right-sided presentation of motor-related problems in PD may have a further asymmetric associate—deficits in olfactory performance—deficits that are contralateral to the presenting side for motor symptoms, but ipsilateral to the side of the brain initially more affected by the disease. This in turn may suggest one contributory factor as to why Doty et al. (1992) may have found no asymmetry in olfactory identification, as their data pooled patients with both bilateral, left and right-sided asymmetries, perhaps obscuring a right-sided effect.

In conclusion, we find that early stage PD patients with right-sided motor symptoms have an associated left-sided deficit in olfactory identification and recognition. That the deficit is ipsilateral to the brain side that is likely to be most affected by PD is not surprising, as olfactory pathways (at least initially) are largely ipsilateral to the presenting nostril (Cain, 1977).

### Conflict of Interest Statement

The authors declare that the research was conducted in the absence of any commercial or financial relationships that could be construed as a potential conflict of interest.
